# LIFESPAN PREDICTION USING MACHINE LEARNING TECHNIQUES ON HISTORIC FULL COUNT CENSUS DATA

**DOI:** 10.1093/geroni/igae098.1753

**Published:** 2024-12-31

**Authors:** Kelsey Carlston

**Affiliations:** Gonzaga University, Spokane, Washington, United States

## Abstract

This study proposes a new measure of status scores for Censuses in the early 1900s. Preceding 1940, income and education were not collected by the US Census, leading researchers to rely primarily on occupation-based metrics such as the IPUMS OCCSCORE or Duncan SEI for continuous status assessment. These measures do not incorporate the rich data available in the Census. Employing machine learning techniques, we aim to develop socioeconomic status scores by leveraging a diverse array of Census variables, including occupation, industry, race, geography, housing tenure, and nativity. The scores will primarily be estimates of life span based on links to Social Security data via CenSoc. The objective of the study is to make publicly available scores that researchers can use in studies of intergenerational mobility, life course studies, and labor force composition, and to determine the most important characteristics from pre-1940 Census data that predict life span.

